# Polyphenols delivery by polymeric materials: challenges in cancer treatment

**DOI:** 10.1080/10717544.2016.1236846

**Published:** 2017-02-03

**Authors:** Orazio Vittorio, Manuela Curcio, Monica Cojoc, Gerardo F. Goya, Silke Hampel, Francesca Iemma, Anna Dubrovska, Giuseppe Cirillo

**Affiliations:** 1 UNSW Australia, Children’s Cancer Institute, Lowy Cancer Research Center and ARC Center of Excellence in Convergent Bio-Nano Science and Technology, Australian Center for NanoMedicine, Sydney, NSW, Australia,; 2 Department of Pharmacy Health and Nutritional Science, University of Calabria, Arcavacata di Rende, Italy,; 3 OncoRay-National Center for Radiation Research in Oncology, Medical Faculty and University Hospital Carl Gustav Carus, Technische Universität Dresden and Helmholtz-Zentrum Dresden-Rossendorf, Dresden, Germany,; 4 Institute of Nanoscience of Aragon (INA) and Department of Condensed Matter Physics, University of Zaragoza, Zaragoza, Spain,; 5 Leibniz Institute of Solid State and Material Research Dresden, Dresden, Germany, and; 6 German Cancer Consortium (DKTK) Dresden and German Cancer Research Center (DKFZ), Heidelberg, Germany

**Keywords:** Nanocarrier, polyphenols, cancer therapy, polymeric materials

## Abstract

Nanotechnology can offer different solutions for enhancing the therapeutic efficiency of polyphenols, a class of natural products widely explored for a potential applicability for the treatment of different diseases including cancer. While possessing interesting anticancer properties, polyphenols suffer from low stability and unfavorable pharmacokinetics, and thus suitable carriers are required when planning a therapeutic protocol. In the present review, an overview of the different strategies based on polymeric materials is presented, with the aim to highlight the strengths and the weaknesses of each approach and offer a platform of ideas for researchers working in the field.

## Introduction

In cancer therapy, the efficacy of conventional cytotoxic agents is dramatically reduced due to their unfavorable physico-chemical and pharmacological properties, consisting in poor water affinity, non-specific distribution within the body, severe toxic effects to healthy cells and tissues, inadequate drug concentrations at tumors sites, and the development of multi-drug resistance (Luo & Prestwich, 2002; Luo et al., 2009; Jemal et al., 2010).

In the last decades, tremendous efforts have been made to overcome these drawbacks by two main approaches:

- Exploration of alternative anticancer agents
- Fabrication of targeted nanocarriers

## Naturally occurring polyphenols: therapeutic opportunities

Among the different bioactive food components with healthy effect on humans, antioxidants and polyphenols in particular are under investigation as both nutraceutical supplement and/or therapeutic agents undergoing intravenous administration (Cirillo et al., 2016).

Polyphenols consist of at least one aromatic ring with one or more hydroxyl groups and differ in terms of number of aromatic rings and number and position of phenolic groups (Bravo, 1998; Nichenametla et al., 2006). They can be preliminarily classified in (Del Rio et al., 2013):

- Flavonoids: 15 carbon atoms with two aromatic rings connected by a three-carbon bridge
- Non-flavonoids: heterogeneous class of compounds, with the C_6_–C_1_ phenolic acids as the most interesting.

**Figure 1.**
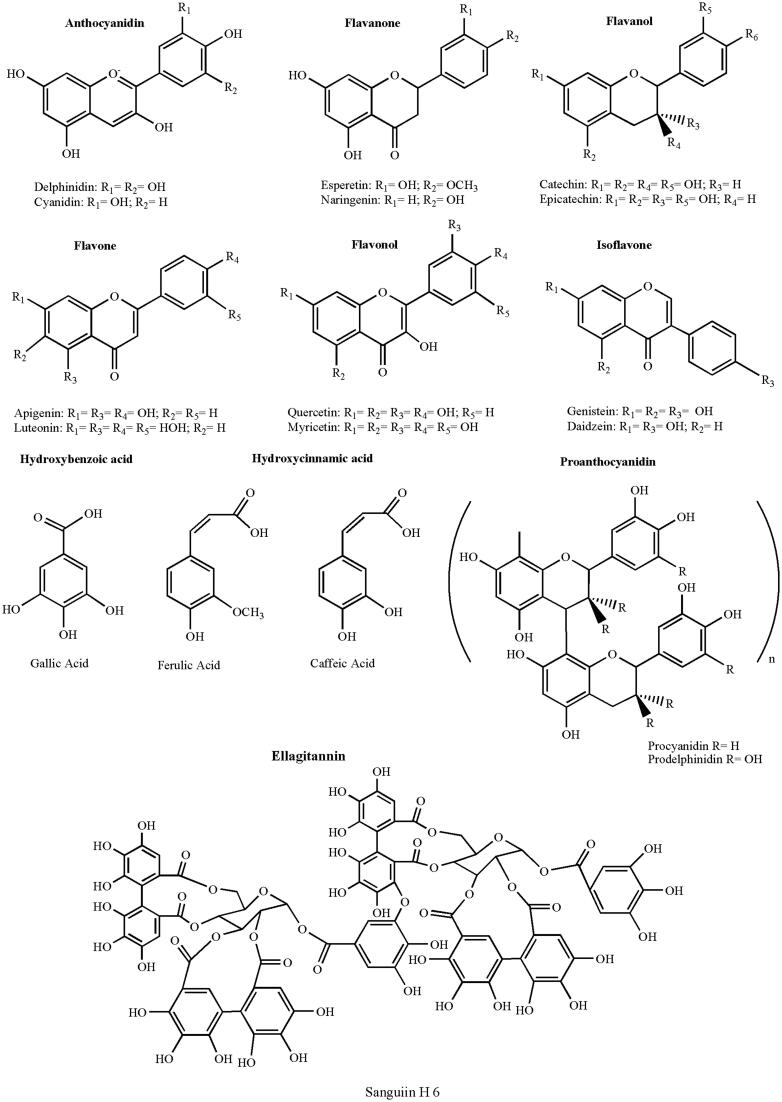
Chemical structure of main polyphenols of biological relevance.

Polyphenols possess different biological activities and have been proposed to exert beneficial effects in a multitude of pathological states, including cancer, cardiovascular, and neurodegenerative diseases (Williams et al., 2004). The antioxidant properties of the polyphenols influencing the intracellular redox status have been hypothesized as the molecular mechanism of action at the basis of their biological functions (Yao et al., 2004). Nevertheless, it was recently speculated that this is unlikely to be the sole explanation for cellular effects (Schroeter et al., 2001; Spencer et al., 2001). It was supposed that they might exert modulatory effects in cells through selective actions on intracellular signaling cascades at different level (Kobuchi et al., 1999; Kong et al., 2000). The structures of polyphenols of biological relevance are sketched in Figure 1 (Dai & Mumper, 2010).

## Anticancer activity of polyphenol: modulation of cell redox state

Extensive laboratory and epidemiological studies have suggested that the cellular damage caused by aberrant production of reactive oxygen species (ROS) such as superoxide, hydrogen peroxide, and hydroxyl radicals is recognized to be the primary cause of aging and age-related diseases, including cancer (Fresco et al., 2006; Hail Jr et al., 2008). Thus, the systematic use of antioxidant molecules to reduce oxidative stress seems to be the ideal approach for cancer prevention (Hou et al., 2005; Khan et al., 2006; Lambert & Elias, 2010). Consistently, extensive epidemiology evidence both in animal and in human studies, suggested that specific dietary antioxidants could be effective in preventing cancer incidence and mortality (Dai & Mumper, 2010). A 5–7% increase in mean survival time and 25–39% decrease in tumor load was recorded in mammary adenocarcinoma bearing mice treated with 0.05% green tea polyphenols as the sole source of drinking fluid, and this was correlated with reduced level of malonyldialdehyde–DNA adduct (a marker of oxidative stress) (Kaur et al., 2007).

Freeze-dried black raspberries were also found to possess high chemoprevention activity in a 6-month chemopreventive pilot study involving patients with Barrett’s esophagus, a premalignant esophageal condition in which the normal stratified squamous epithelium changes to a metaplastic columnar-lined epithelium with increased oxidative stressing conditions (Wang & Sampliner, 2008) by monitoring the urine content, it was proved that high consumption of black raspberries reduced the oxidative stress and the tumor incidence (Kresty et al., 2006). Nevertheless, there are a plenty of research studies with controversial results and a strong debate has started about the real usefulness of polyphenols as therapeutic agents for cancer prevention and/or treatment (Howes, 2006).

**Figure 2.**
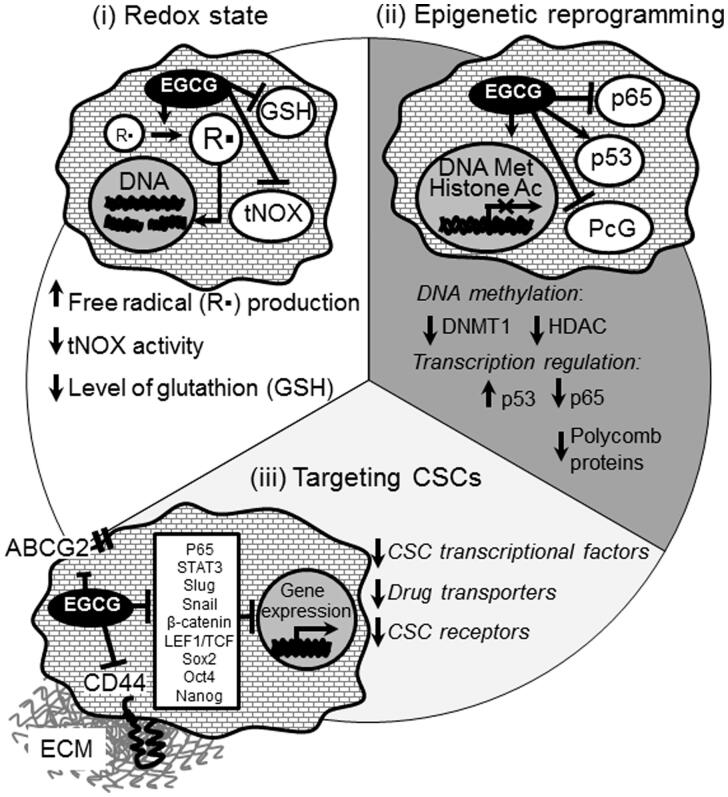
Molecular mechanisms of the biological action of EGCG on tumor cells.

Although the cancer prevention properties of polyphenols are attributed to their anti-oxidant activity, it has been recently shown that the anti-tumor activity arises from their pro-oxidant, rather than antioxidant properties (Hou et al., 2005; Shen et al., 2005) via both direct and indirect mechanisms. Direct pro-oxidant activity involves the formation of liable chemical species (e.g. aroxyl radical and redox complex with a metal cation) under certain conditions, such as high concentration of transition metals and high pH (Procházková et al., 2011; Park & Pezzuto, 2012). Polyphenols can also activate the intracellular production of ROS by indirect mechanisms via interaction with specific cellular pathways (e.g. NOX) (León-González et al., 2015) (Figure 2).

Cancer cells frequently exhibit high oxidative stress, suggesting that it might be possible to preferentially eliminate these cells by pharmacologically increasing the ROS insults (Gupte & Mumper, 2009). However, advanced stage tumor cells developed the ability to adapt to ROS insult by the upregulation of intracellular antioxidant capacity which in turn confers chemo-resistance (Furfaro et al., 2016).

Glutathione (GSH) is an important antioxidant capable of preventing damage to important cellular components caused by reactive oxygen species. Cancer cells usually show elevated GSH level increasing their antioxidant capacity and the resistance to oxidative stress (Ortega et al., 2011). Abrogation of such drug-resistant mechanisms by redox modulation could have significant therapeutic implications. Our recent work has demonstrated that a chemically modified form of polyphenol, called dextran–catechin (Dex–CA), induces oxidative stress in cancer cells and simultaneously increases their sensitivity to intracellular ROS damages by downregulating cellular glutathione. In particular, Dex–CA showed anticancer activity inducing apoptosis in pancreatic ductal adenocarcinoma cells (Vittorio et al., 2012) and was effective in the treatment of neuroblastoma *in vivo* by enhancing ROS generation (Vittorio et al., 2016a). Specifically, the anticancer activity was related to the elevate cellular copper content, which is a common features found in many tumors (Brady et al., 2014). It was demonstrated that neuroblastoma cells display significantly high expression levels of the copper transport 1 (CTR1) and intracellular copper levels resulting in high sensitivity to Dex–CA. In sharp contrast, the depletion of CTR1 expression reduced intracellular copper levels and led to a decrease in neuroblastoma cell sensitivity to the conjugate. In particular, Dex–CA reacts with intracellular copper generating ROS by Fanton reaction (Vittorio et al., 2016a).

In recent studies, the pro-apoptotic effects of the epigallocatechin gallate (EGCG) and other tea polyphenols against H1299 human non-small lung carcinoma (Khan & Mukhtar, 2015) and HL-60 human promyelocytic leukemia cells (Elbling et al., 2005) were related to the induction of ROS production, mainly H_2_O_2_: the co-treatment with exogenous catalase, indeed, resulted in a consistent reduction of EGCG-mediated apoptosis. This confirms that EGCG may undergo intracellular oxidation, and suggest that EGCG-mediated pro-oxidant activity may have anticancer relevance *in vivo*.

Other studies have proved that low concentrations of curcumin promote apoptosis in human cutaneous squamous cell carcinoma by altering of cellular redox homeostasis via depletion of reduced glutathione (Numsen Jr, 2008), and by obstructing mitochondrial function via enhanced ROS production and consequent dissipation of ΔΨm (Scott & Loo, 2004; Atsumi et al., 2006; Su et al., 2006). It was supposed that curcumin promotes the one-electron reduction of superoxide to form the highly biologically reactive hydroperoxyl radical (Mishra et al., 2004; Shen & Ji, 2007) or the reduction of transition metals (e.g. Iron) which in turns catalyze the formation of hydroxyl radical via Fenton chemistry (Galati & O'Brien, 2004; Ligeret et al., 2004; Kawanishi et al., 2005). It is clear indeed that polyphenols play an important role in the regulation of the ROS homeostasis in cancer cells and their combination with standard chemotherapies could potentially overcome drug resistance and improve outcome in patients.

## Anticancer activity of polyphenols: administration of polyphenols for epigenetic reprogramming

Epigenetic modifications are heritable alteration in the structure and function of the chromatin that occur without changes in the DNA sequences. In mammalian cells, these modifications consist of dysregulation of DNA methylation and post-translational histone modifications such as phosphorylation, methylation, acetylation, or ubiquitination, which orchestrate gene expression in various cell types and at the different stages of embryonic development. Furthermore, a view is emerging that epigenetic alteration of the progenitor cells may be an initial step in the development of various diseases including autoimmune diseases, neurodegenerative diseases, and tumor malignancies. In cancer, chromatin reorganization driven by DNA methylation and histone modifications might induce transcriptional silencing of tumor suppressor genes and reactivate transcription of epigenetically silenced oncogenes. A growing body of evidence suggests that epigenetic therapy including DNA-demethylation agents and the drugs targeting histone methylation and acetylation is emerging as an effective and valuable approach for cancer treatment (Yoo & Jones, 2006; Peitzsch et al., 2016; Zahnow et al., 2016). Cancer-specific epigenetic modulators are a unique model for the investigation of cancer development and serve as an attractive therapeutic strategy for cancer treatment. Currently, epigenetic effect of green tea polyphenols such as catechin, curcumin, epigallocatechin-3-gallate (EGCG), and their more stable nano-modified analogs are widely recognized, and their effect on histone modifications and DNA methylation is reported for the various types of cancer cells (Gao & Tollefsbol, 2015). Although the fact that green tea polyphenols such as EGCG are transported to the cell nucleus has been known for a decade, only recently the specific genes in which expression is affected by the polyphenols as well as mechanisms of this regulation appeared in the focus of cancer research (Polster et al., 2003). An effect of the natural polyphenols on the chromatin structure is mediated by inhibition of the key enzymatic proteins involved in DNA and histone modifications such as DNA methyltransferases, histone deacetylases, and histone methyltransferases. Methylation of DNA at the C5-position of cytosine within the CpG islands around the gene promoters is an important mechanism for epigenetic control of gene expression (Gao & Tollefsbol, 2015). Hypomethylation of these CpG sites is associated with gene activation whereas hypermethylation leads to the gene silencing. Recent studies have proven that green tea catechin EGCG reduces methylation of CpG islands within the promoter of tumor suppressor genes p16INK4a, retinoic acid receptor β (RARβ), O6-methylguanine methyltrasferase (MGMT), and human mrtL homolog 1 (hMLH1), and, therefore, reactivates expression of these genes in cancer cells (Fang et al., 2003; Wu et al., 2013). The mechanisms of the EGCG-dependent DNA demethylation are poorly understood. The study of Fang et al. showed that EGCG can inhibit DNA demethylation by forming hydrogen bonds in the catalytic pocket of DNA methyltransferases such as DNMT1 (Lee et al., 2005). The follow-up investigations demonstrated that other dietary polyphenols such as epicatechin, catechin (CA), quercetin (Q), fisetin, and myricetin also have a strong inhibition activity toward DNMT-mediated DNA methylation with IC_50_ values ranged from one to a few μM, but EGCG was the most potent inhibitor among them with IC_50_ value of less than 1 μM (Lee et al., 2005). Epigenetic alteration induced by the natural polyphenols can also be mediated by the inhibition of histone acetyltransferases (HAT) or histone deacetylases (HDAC) by direct binding to the substrate pocket of these enzymes (Choi et al., 2009; Kim & Kim, 2013; Khan et al., 2015). HDACs play an important role in transcription repression by inducing chromatin compaction (Zahnow et al., 2016). Several studies have demonstrated that EGCG-mediated HDAC inhibition is associated with cell-cycle arrest, apoptosis, and metastasis mediated by upregulation of the genes which suppress cancer pathogenesis including Raf kinase inhibitor protein (RKIP) in pancreatic cancer cells, tissue inhibitor of matrix metalloprotease-3 (TIMP3) in breast cancer cell lines, and estrogen receptor α (ESR1) in ER-negative breast tumor cells (Meeran et al., 2012; Kim & Kim, 2013; Deb et al., 2015).

Furthermore, green tea polyphenols can directly activate transcription factors such as tumor suppressor TP53 by the activation of its acetylation that results in increased expression of p21/Waf1 and Bax proteins leading to the growth arrest and apoptosis (Thakur et al., 2012). In contrast, other studies of Choi et al. (2009) demonstrated that EGCG treatment abrogates acetylation of another transcriptional regulator, NFκB-p65, which is mediated by p300/CBP acetyltransferase, that results in suppressing of the NFκB-dependent gene expression and B-cell malignant transformation by Epstein–Barr virus (EBV). Noteworthy, EGCG reduces protein level of some other transcriptional regulators such as polycomb group (PcG) proteins through their proteasome-dependent degradation. PcG proteins are associated with epigenetic gene silencing and implicated in the neoplastic development and metastatic spread (Sparmann & Van Lohuizen, 2006). Treatment of cancer cells with EGCG reduces the level of PcG proteins such as Ezh2, Eed, and Suz12 and decreases the formation of repressive histone marks, H3K27me3 and H2AK119ub (Choudhury et al., 2011).

Taken together, dietary polyphenols might influence the epigenetic landscape and gene expression in cancer cells by several mechanisms that include direct regulation of gene transcription by inhibition of the key enzymes involved in DNA and histone modification, or indirect effect on gene expression by regulation of the multiple transcription factors.

## Biological activity of polyphenols: administration of polyphenols for the targeting of cancer stem cell

Nowadays, the failure of an anticancer treatment is widely attributed to cancer stem cells (CSCs) or tumor initiating cells (TICs), a heterogeneous cell fraction within the tumor which is able to divide asymmetrically and give rise to an identical cell and an intermediate progenitor able to differentiate and repopulate the tumor mass, and is also responsible for chemo and radiotherapy resistance. The capability of these cells to escape treatment has been linked to their specific properties such as increased DNA damage response reflected through higher activation of DNA repair by early triggering of Chk 1 and 2 (Bao et al., 2006), higher levels of anti-apoptotic molecules, and lower levels of the reactive oxygen species (ROS) mediated by the higher presence of free radical-scavenging machinery (Diehn et al., 2009; Cojoc et al., 2015). Together with the above-mentioned intrinsic mechanisms of resistance to therapy, CSCs undergo accumulation of gene alterations such as loss of TP53, RB1, p16, and gain of telomerase, Ras, ERBB2, Braf, c-Myc and WNT/β-catenin signaling mutations (Hanahan & Weinberg, 2011; Baccelli & Trumpp, 2012; Cojoc et al., 2015). The phytochemicals are intensively reported for their cancer preventive and CSCs targeting effects by modulating and regulating multiple self-renewal signaling pathways and transcription factors, fatty acid metabolism and lipid rafts with induction of a ROS-dependent cell proliferation arrest and premature senescence, as well as apoptotic pathway activity.

The green tea polyphenol (GTP) (−)-epigallocatechin-3-gallate (EGCG) has gained considerable attention for its chemo-preventive properties. It has been reported to inhibit nasopharyngeal carcinoma (NPC) CSCs through attenuation of STAT3 (Lin et al., 2014) or NF-κB p65 activity (Li et al., 2015). It has been also shown that synergistic treatment of EGCG with quercetin led to a decrease of CD44+/CD133 + prostate cells through inhibition of Vimentin, Slug, Snail, and nuclear β-catenin expression and LEF-1/TCF responsive reporter activity, affecting the CSC migration and invasion (Tang et al., 2010), and in combination with curcumin, it led to a reduction of sphere formation from breast tumor cells by inhibition of STAT3 phosphorylation and retention of STAT3-NFkB interaction (Chung & Vadgama, 2015). Moreover, in head and neck squamous cell carcinoma (HNSCC), EGCG treatment has been reported to downregulate the expression of stem cell markers, such as Oct4, Sox2, Nanog, and CD44 and to suppress HNSCC cells sphere forming capacity, augmenting their cisplatin (CP)-mediated chemosensitivity by inhibiting ABCC2 and ABCG2 transporter genes (Lee et al., 2013b). In the colorectal tumor model, EGCG induced sensitization of the 5-fluorouracil (5FU)-resistant colorectal cancer cells and spheroid-derived colorectal CSCs to 5FU treatment that was associated with suppression of Notch1, Bmi1, Suz12, and Ezh2, upregulation of self-renewal suppressive-miRNAs, miR-34a, miR-145, and miR-200c and enhancement of apoptosis and cell-cycle arrest (Toden et al., 2016). Catechin is another flavonoid present in a variety of plant sources including grape seeds, tea leaves, apples, apricots, pears, or plums. Catechin is proven to sensitize prostate CSCs to radiotherapy, with reduction of aldehyde dehydrogenase (ALDH) positive fraction, as well as downregulation of stemness factors Nanog and Oct4 (Castro Nava et al., 2016).

Sulforaphane, a dietary component of broccoli or broccoli sprouts, demonstrated its efficacy in targeting CSCs by decreasing of the ALDH positive cell population in human breast cancer cells and reducing the primary mammospheres formation *in vitro* that is accompanied by the downregulation of Wnt/β-catenin self-renewal pathway (Li et al., 2010). The same effect was reported also for the studies on ALDH1 and CD44 positive populations in oral squamous cell carcinomas (OSCCs) where sulforaphane induced miR-200c expression (Liu et al., 2015) and on ALDH1+, CD49f + prostate CSCs with where sulforaphane led to the c-Myc inhibition (Vyas et al., 2016). In the same manner, sulforaphane attenuated expression of pluripotency maintaining transcription factors Nanog and Oct-4 and angiogenic markers VEGF and PDGFRα in human pancreatic CSCs through dysregulation of the sonic hedgehog (Shh)-GLI signaling pathway (Rodova et al., 2012; Li et al., 2013).

The selective efficacy of curcumin and piperine on CSCs, with little or no effect on differentiated cells, has been documented for MCF7 breast tumor cells, where polyphenol treatment led to the inhibition of Wnt signaling pathway and decrease in mamosphere formation by ALDH1 positive cell population (Kakarala et al., 2010). For CD44 positive colorectal CSCs, curcumin might couple with CD44 at the cell membrane with some blocking effect on the transport of glutamine into the cells, decreasing the glutamine content in the CD44(+) cells, and inducing apoptosis (Huang et al., 2016). In H460 lung cancer cells, curcumin was able to reduce the tumor spheres and repress tumor growth in xenograft mouse model via inhibiting the JAK2/STAT3 signaling pathway (Wu et al., 2015).

To β-carotene have been attributed to many anticancer activities, but its exact molecular mechanisms of action on CSCs is not clear yet. Treatment of the neuroblastoma cells with β-carotene led to the induction of neuronal cell differentiation (Lee et al., 2013a) and inhibiting CSCs *in vivo*, while tumor incidence in murine model was significantly inhibited comparing to the untreated group (Lim et al., 2014).

Quercetin, a polyphenol present in many fruit and vegetables, has been studied in different in vitro models for tumor entities like head and neck, where it was able to reduce the ALDH1 positive fraction and inhibit expression of the stemness signatures in head and neck cancer-derived spheres (Chang et al., 2013b) and pancreas cancer, where it chemosensitized the pancreatic CSCs to gemcitabine treatment through β-catenin inhibition (Cao et al., 2015).

The use of soy derivatives has been shown to be beneficial for the reduction of tumor risk for many tumor types. Thus, genistein, a predominant isoflavone found in soy products, has been reported to decrease the CD44/CD24-positive CSC population in MCF7 breast tumor cells and mammosphere formation by downregulating Hedgehog-Gli1 signaling pathway (Fan et al., 2013). Acting on the same signaling pathway in prostate cancer, genistein was shown to suppress prostatosphere and colony formation *in vitro* and tumorigenicity *in vivo* (Zhang et al., 2012a). In gastric cancer cells, genistein was reducing the chemoresistance of CSCs to 5-FU and CP by targeting ABCG2 expression and ERK 1/2 activity (Huang et al., 2014).

The correlation of cancer stem cells and resistance to chemo and radiotherapy encourages the critical consideration for efficient prooxidant CSC-targeted therapy by employing the polyphenols which enable to interfere with the levels of reactive species within these cells, rendering them more sensitive to conventional therapies.

## Nanotechnology and cancer therapy

Cancers originate from alterations in biologic processes at the molecular level, thus strategies acting at the nanoscale are an emerging and promising approach for cancer diagnosis and treatment (Kim et al., 2010; Wang et al., 2012a; Gharpure et al., 2015; Pacardo et al., 2015; Stylianopoulos & Jain, 2015). Different systems have been proposed as drug nanocarriers, including polymeric nanoparticles, vesicles, lipid nanoparticles, nanohybrids, with outstanding results in preclinical research and early clinical studies (Eetezadi et al., 2015; Fernandes et al., 2015; Johnstone et al., 2016).

Nanotherapeutics possess unique properties such as high surface area to volume ratio, and controllable physico-chemical and biological properties via surface functionalization offering solutions to the current obstacles in cancer therapies and allowing the preparation of multiple drug delivery with synergistic therapeutic response or the combination of therapeutic agents and diagnostic probes into theranostic platforms (Kumari et al., 2016). Further advantages related to carrier performance within the physiological environment are the improvement of the therapeutic index, the reduction of the drug toxicity by controlled delivery rate, the improvement of drug’s pharmacokinetic profile by enhanced drug stability and minimized systemic clearance and the obtainment of steady state levels (Kumari et al., 2016). More importantly, the site-specificity of the drug release can be achieved by both, passive (e.g. magnetic field or EPR effect) or active (via suitable surface functionalities) targeting.

These features and benefits can be of dramatic significance for therapeutics agents such as polyphenols whose translation from “bench to bedside” for human use is limited by inefficient systemic delivery, poor bioavailability, and high dosage typically required for optimum response limiting, despite the outstanding advancement in fundamental cancer biology in preclinical settings (Siddiqui et al., 2012). Notably, the majority of *in vitro* studies employ the high concentration of the polyphenols which are not achievable *in vivo* due to their fast degradation caused by the pH or temperature fluctuations. Therefore, development of the nano-modified dietary polyphenols brings a promise to improve their stability and therapeutic performance. Thus, the combination of the favorable biological activity of polyphenols with the technological performance of nanocarriers, overcoming the limitations of conventional chemotherapy at different levels, could represent a fascinating and promising scenario for developing high effective and clinically relevant anticancer therapy.

## Polymeric nanoparticles

Polymeric nanocarriers, defined as colloidal systems with irregular or spherical shape encapsulating or physically entrapping a bioactive molecule (Pérez-Herrero & Fernández-Medarde, 2015), show peculiar properties such as high stability, homogeneous size distribution, controllable physicochemical properties, high drug payload, and controlled drug release (Hu et al., 2010). Different biocompatible polymeric materials from synthetic and/or natural origin with different structure have been employed as nanocarriers in cancer therapy (Tsouris et al., 2014; Estanqueiro et al., 2015). Some key examples of nanoparticulate polymer systems proposed for the delivery of polyphenols are summarized in Table 1. The main attention is devoted to the materials coupling biocompatibility and biodegradability such as naturally occurring chitosan (Jayakumar et al., 2010) and synthetic PLGA (Danhier et al., 2012), while PEG derivatives are widely explored for the surface functionalization preventing macrophage uptake of nanoparticles (Dong et al., 2015).

**Table 1.** Composition and activity of main polyphenol nanocarriers employed in cancer therapy.

			Polyphenol					
Carrier					Anticancer activity			
Type	Composition	Preparation	Type	Cancer type	Cells	Synergism	*In vivo* model	References
NPs	PLGA-PEG	ESE	PmPs	Breast	MCF-7, Hs578T	–	–	Shirode et al. (2015)
NPs	CT	IG	TPs	Hepatic	HepG2	–	–	Liang et al. (2014)
NPs	CT	IG	TPs	Hepatic	HepG2	–	–	Liang et al. (2011)
NF	PCL	ESP	PPs	Gastric	MKN28	–	–	Kim et al. (2012)
NPs	ALG-CT-PX F127	IG	CUR	Cervice	HeLa	–	–	Das et al. (2010)
NPs	PLGA	ESE	CUR	Oral	CAL27	–	–	Chang et al. (2013a)
NPs	FB	CaCl_2_ CK	CUR	Prostate	PC3	–	–	Sanoj Rejinold et al. (2011)
				Breast	MCF-7			
NPs	PLGA	ESE	CUR	Prostate	LNCaP, PC-3, DU-145	–	–	Mukerjee & Vishwanatha (2009)
NPs	PLGA	ESE	CUR	Osteosarcoma	U2OS	–	–	Peng et al. (2014)
HYs	CHT	IG	CUR	Melanoma	A375	–	–	Mangalathillam et al. (2012)
NPs	Peptide	SA	CUR	Medulloblastoma	DAOY	–	–	Altunbas et al. (2011)
NPs	NIPAA-VP-PEG-MA	RP	CUR	Pancreas	MiaPaCa2, Su86.86, BxPC-3, Capan-1, PANC-1, E3LZ10.7, PL-5, PL-8	–	HM	Bisht et al. (2007)
NPs	CT-PBCA	EP	CUR	Hepatic	HepG2, Bel7402, Huh7	–	HepG2 XM	Duan et al. (2010)
HYs	PX 407–PX F188	SA	CUR	Melanoma	B16-F10	–	–	Sun et al. (2014b)
NPs	CL	NPR	CUR	Prostate	C4-2, LNCaP, DU-145, PC-3	–	–	Yallapu et al. (2012)
NPs	PLGA	ESE	CUR	Cervice	HeLa	–	–	Nair et al. (2012)
NPs	PLGA–PX F127	NPR	CUR	Cervice	KB-V1, KB-3-1	–	–	Punfa et al. (2012)
HYs	PEG-DA-AA	RP	CUR	Cervice	HeLa	–	–	Deepa et al. (2012)
HYs	FA-PEG-DA-AA	RP	CUR	Cervice	HeLa	–	–	Pillai et al. (2014)
NPs	PLGA	NPR	CUR	Ovarian	A2780, A2780CP	–	–	Yallapu et al. (2010a)
NPs	PLGA-PEG	ROP	CUR	Breast	MCF-7	–	–	Mirakabad et al. (2016)
NPs	PLGA	ESE	CUR	Breast	MCF-7	–	–	Verderio et al. (2013)
NPs	EU-S100	ESE	CUR	Colon	HT-29	–	–	Prajakta et al. (2009)
NPs	PLGA-PEG	NPR	CUR	Colon	HT29	–	HM	Li et al. (2014)
NPs	HSA	ESE	CUR	Colon	HCT116	–	HCT116 XM	Kim et al. (2011)
				Pancreas	MiaPaCa2			
HYs	CT, GL, HA	EFS	CUR	Lung	A549	–	–	Teong et al. (2015)
NPs	NIPAA-VP-AA	RP	CUR	Medulloblastoma	DAOY, D283Med	–	–	Lim et al. (2011)
				Glioblastoma	HSR-GBM1, JHH-GBM14			
HYs	CT	IG	CUR	Oral	SCC25	–	–	Popat et al. (2014)
NPs	PLGA	NPR	CUR	Prostate	DU-145, PC-3	–	XM	Yallapu et al. (2014)
HYs	GL-PAGA	EP	CUR	Colon	HCT-116	–	–	Madhusudana Rao et al. (2015)
NPs	PLGA	ESE	CUR	Breast	MCF-7	5-FU	–	Balasubramanian et al. (2014)
NPs	CT-PBCA	EP	CUR	Breast	MCF-7	DOX	–	Duan et al. (2012)
NPs	PLGA	NPR	CUR	Ovarian	A2780CP	CP	–	Yallapu et al. (2010b)
				Breast	MDA-MB-231			
NPs	NIPAA-VP-AA	RP	CUR	Pancreas	–	GM	Pa03C XM	Bisht et al. (2010)
NPs	GL-PEC	LbL	EGCG	Breast	MBA-MD-231	–	–	Shutava et al. (2009)
NPs	CS-pp-CT	GP-CK	EGCG	Hepatic	HepG2	–	–	Hu et al. (2014)
				Gastric	BGC823			
NPs	CS-pp-CT	GP-CK	EGCG	Colon	Caco-2	–	–	Hu et al. (2015)
NPs	PLGA-PEG	NPR	EGCG	Prostate	LNCaP	–	–	Sanna et al. (2011)
NPs	HA	SA	EGCG	EAC	EAC	DOX	–	Ray et al. (2013)
NPs	CT	IG	EGCG	Melanoma	Mel928	–	Mel928 XM	Siddiqui et al. (2014)
NPs	PLGA-PEG	NPR	EGCG	Prostate	PC-3	–	22Rυ1 XM	Siddiqui et al. (2009)
NPs	CT	IG	EGCG	Prostate	–	–	22Rυ1 XM	Khan et al. (2014)
NPs	CT-GL-PEG	IG	EGCG	Gastric	Luc MKN45	–	XM	Lin et al. (2015)
NPs	PLGA	NPR	EGCG	Lung	A549	CP	–	Singh et al. (2011)
				Cervice	HeLA			
			TF	Leukemia	THP-1			
NPs	PLGA	SE	EGCG	Lung	A549	CP	EAC XM	Singh et al. (2015)
				Cervice	HeLA			
			TF	Leukemia	THP-1			
				EAC	–			
NPs	BSA	NPR	RV	Lung	NCI-H460	–	–	Karthikeyan et al. (2015)
NPs	BSA	NPR	RV	Ovarian	SKOV3	–	–	Guo et al. (2015)
NPs	PLGA	EM	RV	Breast	MCF-7	–	–	Kumar et al. (2016)
NPs	PLGA-PEG	NPR	RV	Prostate	DU-145, LNCaP	–	–	Sanna et al. (2013)
NPs	MI-PEG-PLA	SA	RV	Glioblastoma	CT26, U87	–	CT26 XM	Guo et al. (2013)
NPs	CT	IG	Q	Pancreas	MiaPaCa2,	5-FU	–	David et al. (2015)
NPS	PLGA	ESE	Q	Breast	MCF-7	TM	XM	Jain et al. (2013)
				Colon	Caco2			
NPs	HA- PBCA-α-TPh-PEG-SC	RP	MH	Lung	A549	–	S180 XM	Abbad et al. (2015)
				Hepatic	L02			
PC	PEG	CM	CUR	Glioma	C6	–	–	Dey et al. (2015)
PC	PEG	CM	CUR	Prostate	PC-3	–	–	Safavy et al. (2007)
PC	PEG	CM	CUR	Pancreas	MiaPaCa2, PANC-1, BxPC-3, AsPC-1	GM	–	Li et al. (2009)
PC	PEG	CM	RV	Cervice	HeLa	BT	–	Wang et al. (2016)
				Breast	MCF-7			
PC	CMCT	CM	Q	Hepatic	HepG2	PTX	HepG2 XM	Wang et al. (2014)
PC	PEG	CM	CUR	Cervice, Breast	HeLa, EMT6	–	EMT6 XM	Lv et al. (2015)
PC	PEG-DTE	CM	CUR	Breast	MDA-MB-231	–	–	Shpaisman et al. (2012)
PC	PEG	CM	CA	Breast	MDA-MB-231	BZ	–	Su et al. (2011)
PC	HA-PEI	CM	EGCG	Colon	HCT-116	GB	–	Liang et al. (2016)
PC	Dex	FRG	CA	Pancreas	MiaPaca-2, PL45	–	–	Vittorio et al. (2012)
PC	Dex	FRG	CA	Neuroblastoma	IMR-32, IMR-32-CisRes, BE(2)-C	–	XM	Vittorio et al. (2016a)
PC	Dex	ELC	CA	Neuroblastoma	IMR-32	–	–	Vittorio et al. (2016b)
PC	GL	FRG	GA	Prostate	DU-145, PC-3	–	–	Cirillo et al. (2010)
				Kidney	A498			
PC	PMAA	FRG	Q	Cervice	HeLa	–	–	Puoci et al. (2012)
MCs	GL-Dex	SA-GP-CK	PPs	Breast cancer	MCF-7	–	–	Zhou et al. (2012)
PMs	PVP-PEG	EE	PPs	Glioblastoma	DBTRG-05MG	–	–	Wang et al. (2015a)
MCs	GL-Dex	SA-GP-CK	CUR	Cervice	HeLa	–	HM	Zhang et al. (2014a)
PMs	KT	SE	CUR	Cervice	HeLa	–	–	Curcio et al. (2015a)
PMs	GL	Se	CUR	Lung	H1299	–	–	Curcio et al. (2015b)
MCs	CS	SA	CUR	Cervice	HeLa	–	–	Sahu et al. (2008b)
MCs	ZN-PEG	SA	CUR	Ovarian	NCI	–	HM	Podaralla et al. (2012)
MCs	CT-STA	SA	CUR	Colon	Primary	–	XM	Wang et al. (2012b)
MCs	PEG-PAE	SE	CUR	Cervice	HeLa	–	–	Lv et al. (2013)
MCs	PEG-PLA	SE	CUR	Breast	MCF-7	DOX	XM	Lv et al. (2016)
MCs	PEG-PCL	TLE	CUR	Ovarian	A2780t	–	–	Gou et al. (2015)
MCs	PEG-PCL	TLE	CUR	Breast	MDA-MB-436	–	–	Zhu et al. (2015)
MCs	PEG-PCL	SA	CUR	Breast	4T1	–	4T1 XM	Liu et al. (2013)
MCs	PEG-PCL	TLE	CUR	Cervice	HeLa	–	XM	Mikhail et al. (2014)
				Colon	HT-29			
MCs	PEG-PCL	TLE	CUR	Lung	LL/2	DOX	XM	Wang et al. (2013)
MCs	PEG-PCL	SA	CUR	Lung	LL/2	–	ZF	Gong et al. (2013)
MCs	LNA-PEG-PCL	SA	CUR	Cervice	HeLa	–	HM	Song et al. (2014)
				Lung	A549			
MCs	PEG -PAC	SA	CUR	Cervice	HeLa	–	–	Sahu et al. (2008a)
PMs	PEG-OA	TLE	CUR	Brain	U87MG	–	–	Erfani-Moghadam et al. (2014)
MCs	PEG2000-DSPE	TLE	CUR	Ovarian	SK-OV-3TR	PTX	–	Abouzeid et al. (2014)
MCs	PEG2000-DSPE	TLE	CUR	Ovarian	NCI	PTX	SK-OV-3TR XM	Sarisozen et al. (2014)
MCs	PEG2000-DSPE	TLE	CUR	Colon	HCT-116	DOX	XM	Abouzeid et al. (2013)
MCs	PEG-DOX	SA	CUR	Cervice	HeLa	DOX	HepG2 XM	Zhang et al. (2016)
				Hepatic	HepG2			
MCs	PX-PEG-SC	SE	CUR	Ovarian	NCI	–	–	Saxena & Hussain (2013)
MCs	PX F127 F68	TLE	CUR	Cervice	HeLa	–	–	Sahu et al. (2011)
MCs	PX F127	TLE	RV, CUR	Ovarian	SKOV-3	DOX	HM	Carlson et al. (2014)
MCs	PX F127	TLE	RV, Q	Ovarian	SKOV-3	DOX	HM	Cote et al. (2015)
MCs	PCL-PEG-SC	TLE	RV	Breast	MCF-7	–	–	Wang et al. (2015b)
MCs	AP-E3	rDNA	RV	Glioblastoma	A-172	–	–	Kim et al. (2015a)
MCs	PLA-PEG	TLE	EGCG	Pancreas	MiaPaca-2	–	–	Sun et al. (2014a)
MCs	CS	SA	EGCG	Colon	HT-29	–	–	Haratifar et al. (2014b)
MCs	CS	SA	EGCG	Colon	–	–	XM	Haratifar et al. (2014a)
CNs	GO	RM	TPs	Colon	HT29, SW48	–	–	Abdolahad et al. (2013)
CNs	GO	RM	RV	Ovarian	A2780	–	–	Gurunathan et al. (2015)
CNHs	PCL-MWNT	ESP	TPs	Lung	A549	–	–	Shao et al. (2011)
				Hepatic	HepG2			
CNHs	GL-MWNT	CG	CA	Prostate	DY-145, PC-3, LNCap	RT	–	Castro Nava et al. (2016)
CNHs	GL-MWNT	CG	CA	Cervice	HeLa	–	–	Cirillo et al. (2013b)
CNHs	PMAA-MWNT	RC	Q	Cervice	HeLa	–	–	Cirillo et al. (2013a)
CNHs	PMAA-MWNT	RC	Q	Neuroblastoma	IMR-32	CP	–	Vittorio et al. (2014a)
MNPs	Fe-CA	CPM	CUR	Breast	MCF-7	–	–	Wani et al. (2011)
MNPs	HA-Fe	LbL	CUR	Colon	Caco-2	–	–	Manju & Sreenivasan (2011)
	PVP-Fe			Glioma	C6			
MNPs	Fe-PX F127	NPR	CUR	Pancreas	HPAF-II, Panc-1	–	XM	Yallapu et al. (2013)
MNPs	Fe-DEX	SM	CA	Pancreas	MIA Paca2	–	–	Vittorio et al. (2014b)
MNPs	Fe	KL	EGCG	Colon	CT-26	–	XM	Xiao et al. (2015)
MNPs	Ni	ED	Q	Hepatic	SMMC-7721	–	–	Guo et al. (2009)

PEG2000-DSPE, 1,2-distearoyl-sn-glycero-3-phosphoethanolamine-N-[methoxy(polyethylene glycol)-2000]; 5-FU, 5-fluorouracil; AA, acrylic acid; ALG, alginate; AP, apolipoprotein; BT, bicalutamide; BZ, bortezomib; BSA, bovine serum albumin; CPM, capping method; CNHs, carbon nanohybrids; CNs, carbon nanostructures; CMCT, carboxymethyl chitosan; CS, casein; CA, catechin; CL, cellulose; KM, chelation method; CHT, chitin; CT, chitosan; CP, cisplatin; CG, coating; CM, condensation method; CK, crosslinking; CUR, curcumin; DTE, desaminotyrosyl-tyrosine ethyl ester; Dex, dextran; DOX, doxorubicin; EAC, Ehrlich ascites carcinoma; ED, electrochemical deposition; ESP, electrospinning; EFS, electrostatic field system; ESE, elmusion solvent evaporation; EE, emulsion evaporation; EM, emulsion method; EP, emulsion polymerization; ELC, enzyme laccase catalysis; EGCG, epigallocatechin gallate; EU, eudragit; FB, fibrinogen; FA, folic acid; FRG, free radical grafting; GA, gallic acid; GL, gelatin; GM, gemcitabine; GP, genipin; GB, granzyme B; GO, graphene oxide; GG, guar gum; HM, healthy mice; HSA, human serum albumin; HA, hyaluronic acid; HYs, hydrogels; IG, ionic gelation; KT, keratin; LbL, layer-by-layer; LNA, linoleic acid; MNPs, magnetic nanoparticles; MI, maleimide; MCs, micelles; MH, morin hydrate; MWNT, multi-walled carbon nanotubes; NFs, nanofibers; NPs, nanoparticles; NPR, nanoprecipitation; NIPAA, N-isopropylacrylamide; VP, *N*-vinyl-2-pyrrolidone; OA, oleic acid; PTX, paclitaxel; PAC, palmitic acid; pp, phosphopeptide; PPs, plant polyphenols; PX, poloxamers; PAGA, poly(acrylamidoglycolic acid); PA, poly(acrylates); PAE, poly(anhydride esters); PBCA, poly(butyl cyanoacrylate); PCL, poly(caprolactone); PCA, poly(cyanoacrylates); PEC, polyelectrolyte; PEG-mA, poly(ethylene glycol) acrylate; PEG-DA, poly(ethylene glycol) diacrylate; PEG, poly(ethylene glycols); PEI, poly(ethyleneimine); PLA, poly(lactic acid); PLGA, poly(lactide-*co*-glycolide) acid; PMAA, poly(methacrylic acid) ; PVP, poly(vinyl pyrrolidone); PC, polymer conjugate; PMs, polymersomes; PmPs, pomgranade polyphenols; Q, quercetin; RC, radical coupling; RP, radical polymerization; RT, radiotherapy; rDNA, recombinant DNA; RM, reduction method; RV, resveratrol; ROP, ring opening polymerization; SA, self-assembly; SM, solvation method; SE, solvent evaporation; STA, stearic acid; SC, succinate; TM, tamoxifen; TPs, tea polyphenols; TF, theaflavin; TLE, thin layer evaporation; TPh, tocopheryl; XM, xenograft mice; ZF, zebrafish; ZN, Zein.

Different synthetic strategies have been proposed for the preparation of effective delivery vehicles, depending on the physico-chemical properties of the starting polymer, mainly retained in the final product, and on the desired technological features of the carriers effecting their performance both *in vitro* and *in vivo* experiments. The widely explored methodologies involved polar interaction, emulsion solvent evaporation, and radical polymerization (Table 1).

*In vitro* experiments showed that the loading into Chitosan (CT), poly(caprolactone) (PCL), and poly(lactide-*co*-glycolide) acid (PLGA) nanoparticles carried out to the enhancement of the antiproliferative effects of polyphenols extracts through effective cell internalization (Shirode et al., 2015), necrosis and/or apoptosis induction in hepatic (Liang et al., 2011,2014), gastric (Kim et al., 2012), and breast (Shirode et al., 2015) cancer cell lines with superior compared with their free counterparts.

As before mentioned, curcumin (CUR) is one of the most promising polyphenols with biological activity, and several efforts have been performed in pointing out an effective formulation overcoming its poor water solubility. The most of the proposed delivery nano-systems show mechanisms of action mirror that of free curcumin, allowing an effective passive targeting on different cancer cells, including cervical (Das et al., 2010), oral (Chang et al., 2013a), prostate (Mukerjee & Vishwanatha, 2009; Sanoj Rejinold et al., 2011), breast (Sanoj Rejinold et al., 2011) cancers, osteosarcoma (Peng et al., 2014), melanoma (Mangalathillam et al., 2012), and medulloblastoma (Altunbas et al., 2011) by controlling the CUR release over time. Furthermore, *in vivo* studies proved that nanoparticles prepared by free radical polymerization of *N*-isopropylacrylamide (NIPAAm), *N*-vinyl-2-pyrrolidone (VP), and poly(ethylene glycol) acrylate (PEG-mA), proposed for the treatment of pancreatic cancer, show negligible toxicity in mouse model (Bisht et al., 2007), while the emulsion polymerization of butyl-cyanoacrylate in the presence of CT allows the obtainment of a CUR delivery vehicles suitable for the treatment of hepatic cancer with favorable pharmacokinetic profiles (Duan et al., 2010).

In addition to these features, improved cellular uptake and/or apoptosis induction by CUR nanoformulations result in enhanced anticancer activity in melanoma (Sun et al., 2014b), prostate (Yallapu et al., 2012), cervical (Deepa et al., 2012; Nair et al., 2012; Punfa et al., 2012; Pillai et al., 2014), ovarian (Yallapu et al., 2010a), breast (Yallapu et al., 2010a; Verderio et al., 2013; Mirakabad et al., 2016), colon (Prajakta et al., 2009; Li et al., 2014), pancreatic (Kim et al., 2011), lung (Teong et al., 2015) medulloblastoma (Lim et al., 2011), glioblastoma (Lim et al., 2011), and oral carcinoma (Popat et al., 2014) cancer cell lines with superior pharmacokinetics (Li et al., 2014). Furthermore, PLGA-PEG nanoparticles are able to enhance the anticancer efficiency of CUR in prostate (Yallapu et al., 2014) and colon (Kim et al., 2011) cancer cell both *in vitro* and *in vivo*. The enhancement of CUR activity was also reached by encapsulation into GL-based nanogels able to target the deliver the polyphenol to colon cancer due to pH responsivity (Madhusudana Rao et al., 2015).

CUR nanocarriers have been also proposed for the treatment of breast cancer in combination therapy with conventional cytotoxic agents such as 5-FU (Balasubramanian et al., 2014), Doxorubicin (DOX) (Duan et al., 2012), and CP (Yallapu et al., 2010b) with synergistic activity. The association with CP is also useful for ovarian cancer treatment (Yallapu et al., 2010b), while *in vivo* studies proved the synergism of CUR nanoformulation prepared by free radical polymerization of NIPAAm, VP, and AA with Gemcitabine in Pa03C Xenograft mouse model (Bisht et al., 2010).

EGCG stability in physiological environments and *in vitro* anticancer activity against breast (Shutava et al., 2009), hepatic (Hu et al., 2014), gastric (Hu et al., 2014), colon (Hu et al., 2015), and prostate cancer (Sanna et al., 2011) were improved in nanoparticulate systems based on naturally occurring polymers, including (gelatin – GL) (Shutava et al., 2009), casein–chitosan (CS–CT) derivative (Hu et al., 2014,2015), or synthetic PLGA-PEG derivative (Sanna et al., 2011). Synergistic effect was recorded by using EGCG-hyaluronic acid (HA) nanoparticles in combination with DOX for the treatment of Ehrlich ascites carcinoma (EAC) (Ray et al., 2013).

The effective suitability of EGCG as anticancer agent was proved by *in vivo* experiments involving EGCG nanoformulations and xenograft mice models of melanoma (Siddiqui et al., 2014), prostate (Siddiqui et al., 2009; Khan et al., 2014) and gastric (Lin et al., 2015) cancers. Furthermore, the *in vitro* (Singh et al., 2011), and *in vivo* (Singh et al., 2015) synergism recorded when EGCG was delivered in association with CP, offers new therapeutic opportunities for some invasive human cancer diseases.

Resveratrol (RV) was effectively released from GL (Karthikeyan et al., 2015), Bovine Serum Albumin (BSA) (Guo et al., 2015), PLGA (Kumar et al., 2016), PLGA-PEG (Sanna et al., 2013), nanoparticles, showing enhanced anticancer efficiency for the treatment of lung (Karthikeyan et al., 2015), ovarian (Guo et al., 2015), breast (Kumar et al., 2016), and prostate (Sanna et al., 2013) cancers. The insertion of transferrin units on PLGA-PEG nanoparticles allows the obtainment of RV nanovehicles with favorable pharmacokinetic profiles and active targeting effect on glioma cancer cells *in vivo* (Guo et al., 2013).

When Q is loaded on CT nanoparticles show synergistic effect with 5-FU towards pancreatic cancer cells both in the 2D and in the 3D cultures (David et al., 2015), while its encapsulation into PLGA was suitable for the treatment of breast cancer in xenograft mouse model (Jain et al., 2013).

Finally, a hyaluronic acid-polybutyl cyanoacrylate-α-tocopheryl-PEG-succinate (HA-PBCA-α-TPh-PEG) carrier system was proposed as delivery vehicle for the treatment with MH of lung and hepatic cancer *in vitro* and sarcoma *in vivo* (Abbad et al., 2015).

## Polymer conjugates

Polymer conjugates, composed of a drug covalently linked to a water-soluble macromolecular system, are emerging tools as anticancer therapeutics, and relevant research activity are exploring their impact in novel and performing therapies. The high molecular weight confers the same favorable pharmacokinetic and toxicological features discussed for polymeric nanoparticles, and the ability to overcome some mechanisms of drug resistance (Minko et al., 1998) and to elicit immunostimulatory effects (Říhová et al., 2003; Sirova et al., 2013).

According to the Ringsdorf model (Ringsdorf, 1975), a water-soluble polymeric–drug conjugate consist of three different units (Sobczak et al., 2013): the drug-linking portion, a moiety responsible for the physicochemical properties; a third unit incorporating an active targeting element (e.g. monoclonal antibody) allowing site-specificity at the cellular level (Allen, 2002). The concept of polymer therapeutics can be extended to antioxidant polymers, obtained either by the polymerization of monomeric polyphenols or their conjugation to natural or synthetic macromolecules. Three main approaches have been proposed for the synthesis of high molecular weight antioxidants, namely condensation, radical grafting, and enzymatic catalysis (Spizzirri et al., 2014).

The condensation methods involve reactions between the chemical functionalities within the antioxidant molecules and those inside the polymeric backbones (e.g. acylation, esterification, etc.), allowing the possibility to modulate the properties of the final products (e.g. mechanical, physical, etc.). The methods are very versatile in terms of substrate availability (e.g. chemical composition and type of the coupling linkage), but the overall reaction process is often a multi-step reaction and is mainly employed in the case of functionalization of synthetic polymers, since the mechanical properties remain almost unchanged compared to the parent material.

The radical grafting approach works in mild reaction conditions almost completely preserving the chemical integrity of the polyphenol moiety and can be applied to polymeric materials (e.g. natural polymers like proteins and polysaccharides) possessing chemical functionalities able to undergo free radical coupling (e.g. heteroatoms and/or aromatic rings) (Oliver et al., 2016).

Finally, the third approach involves a coupling reaction in the presence of a suitable enzyme (laccases, peroxidases, or tyrosinases) as a catalyst and allows synthesizing well-defined structures with controlled stereo- and chemo-selectivity in milder reactions conditions (temperature, pressure, pH) and without using toxic reagents (Nyanhongo et al., 2012; Ravichandran et al., 2012; Zhang et al., 2012b).

Curcumin conjugates have been synthesized by condensation reaction with PEG via carbodiimide chemistry, and proposed as a polymer therapeutics for the treatment of glioma (Dey et al., 2015), prostate (Safavy et al., 2007), and pancreatic cancer in combination with gemcitabine (Li et al., 2009). Carbodiimide was also employed for the preparation of PEG-RV conjugate showing cytotoxic activity in cervical and breast cancer cells in synergism with Bicalutamide (Wang et al., 2016), and carboxymethyl chitosan-Q conjugate for the treatment of hepatic cancer *in vitro* and *in vivo* in combination with Paclitaxel (PTX) (Wang et al., 2014). An interesting upgrade of carbodiimide chemistry was proposed in (Lv et al., 2015), where CUR was at first reacted with dithiopropionic acid, and the resulting hydrophobic co-polymer was conjugated with PEG in the presence of biotin as targeting element. Then, emulsion solvent evaporation method allows the obtainment of nanoparticles employed for the treatment of cervical (*in vitro*) and breast (*in vitro* and *in vivo*) cancers in synergism with DOX. A different approach for CUR conjugation was proposed when triphosgene chemistry was employed for the preparation of CUR-PEG hydrogels with enhanced anti-proliferative activity in breast cancer cells (Shpaisman et al., 2012).

By amidation with PEG (Su et al., 2011) and hyaluronic acid (Liang et al., 2016), the anticancer activity of CA and EGCG against breast and colon cancer was significantly enhanced in synergism with Bortezomib and Granzyme B, respectively. The conjugation of CA to Dex by free radical (Vittorio et al., 2012, 2016a) or laccase catalysis (Vittorio et al., 2016b) allows the obtainment of polymer conjugates suitable for the treatment of pancreatic cancer *in vitro* (Vittorio et al., 2012) and neuroblastoma in vivo (Vittorio et al., 2016a) with higher efficiency when compared with the free flavonoid. A free radical approach was also explored for the synthesis of a GL–gallic acid (GL–GA) (Cirillo et al., 2010) and polymethacrylic acid-Quercetin (PMAA-Q) (Puoci et al., 2012) conjugates showing anticancer activity towards prostate and cervical cancer, respectively.

## Polymeric vesicular systems

Amphiphilic block copolymers, composed of a hydrophobic and a hydrophilic portion, are able to form self-assembled structures with different size and shape, including spherical micelles (Discher et al., 2000), worm-like micelles, and closed bi- or multilayer structures (Kumar et al., 2007), named polymersomes (Brinkhuis et al., 2011). Both micelles and polymersomes are promising candidate for drug delivery applications, offering some peculiar advantages.

Polymeric micelles can delivery both water soluble and insoluble molecules by encapsulation in the hydrophobic core or inclusion in the hydrophilic shell (Onaca et al., 2009). They show reduced size (20–80 nm), long circulation times in the bloodstream (Tong & Cheng, 2007; Torchilin, 2007), while suffer from insufficient stability in systemic circulation and premature drug leakage which may cause side effects and a decrease of effectiveness (Lu & Park, 2013).

Polymersomes are bilayered systems with size and shape close to liposomes but showing key advantages for the modulation of the delivery of an encapsulated drug related to the lower permeability since they are composed of amphiphiles with higher molecular weight and conformational freedom, the higher mechanical stability due to the thicker membrane, and the possibility to tune the membrane properties through the polymer chemistry (Onaca et al., 2009). In addition, the interaction of polymersomes with macrophages is reduced due to the conveying surface-protective properties of the employed shell-forming hydrophilic flexible polymers (Brož et al., 2005).

As previously discussed, the use of biodegradable or at least biocompatible polymers is obligatory for biomedical applications, thus polymeric micelles and polymersomes suitable for polyphenol delivery are formed by naturally occurring polysaccharides (e.g. Dex and CT), proteins (e.g. GL, CS) of synthetic building blocks mainly composed of PEG, PCL, PX, and their derivatives (e.g. fatty acid esters), see Table 1.

Plant polyphenols were successfully encapsulated into GL-Dex (Zhou et al., 2012) micelles and PVP-PEG (Wang et al., 2015a) polymersomes and explored *in vitro* for the treatment of breast cancer with higher efficiency than their free counterpart (Zhou et al., 2012) and glioblastoma by caspase-dependent activation of both the intrinsic and extrinsic signaling pathways (Wang et al., 2015a).

The imprinted GL-Dex micelles prepared in the presence of tea polyphenols (Zhou et al., 2012) were also proposed as nanocarriers for CUR and tested for the treatment of HeLa cancer cells (Zhang et al., 2014a). Interestingly, such vehicle improved the CUR pharmacokinetics profile with considerable therapeutic benefits.

Proteins from both animal (keratin – KT, GL, and CS) and vegetal (Zein) sources were explored as starting materials for the preparation of CUR delivery devices by solvent evaporation (Curcio et al., 2015a,b) and self-assembly (Sahu et al., 2008b; Podaralla et al., 2012). The main advantages of such materials are the high biocompatibility and improved CUR efficiency on cervical (Sahu et al., 2008b; Curcio et al., 2015a) and lung (Curcio et al., 2015b) cancer cells *in vitro* and ovarian cancer with negligible immunogenicity in mice (Podaralla et al., 2012). Amphiphilic behavior was conferred to CT by conjugation with STA, allowing the obtainment of CUR carrier for the treatment of colon cancer both *in vitro* (primary cancer cells) and *in vivo* (orthotopic mice) with improved efficiency (Wang et al., 2012b). The high anticancer efficiency of CUR is responsible for the wide researches devoted to the preparation of micelles and polymersomes able to enhance its performance. PEG is the synthetic polymers at the basis of most of the proposed vesicular delivery systems, due to its biocompatibility and stealth properties. By solvent evaporation, PEG in combination with poly(anhydride esters) (PAE) (Lv et al., 2013) and poly(lactic acid) (PLA) (Lv et al., 2016) carried out to micelles employed as CUR vehicle in HeLa (Lv et al., 2013) *in vitro* and in MCF-7 cancer cells with considerable results in xenograft mouse model in synergism with DOX (Lv et al., 2016). In different studies including *in vitro* (Gou et al., 2015) or *in vivo* cancer models, PEG-PCL micelles prepared by self-assembly or thin layer evaporation were proposed for the treatment of ovarian (Gou et al., 2015) and breast (Zhu et al., 2015) cancer cells, breast (Liu et al., 2013), cervical (Mikhail et al., 2014), colon (Mikhail et al., 2014), and lung (Gong et al., 2013; Wang et al., 2013) xenograft mouse model, showing anti-angiogenesis properties in zebrafish (Gong et al., 2013; Wang et al., 2013). A further modification of PEG-PCL micelles by introduction of fatty acids residues (e.g. linoleic acid – LNA, palmitic acid – PAC, and oleic acid – OA) allows the obtainment of CUR delivery nanocarriers suitable for the treatment of cervical (Sahu et al., 2008a; Song et al., 2014), brain (Erfani-Moghadam et al., 2014), and lung tumors (Erfani-Moghadam et al., 2014), with superior efficiency and pharmacokinetics (Song et al., 2014). A different approach involves the employment of 1,2-distearoyl-sn-glycero-3-phosphoethanolamine-*N*-[methoxy(polyethylene glycol)-2000] in a thin-layer evaporation methods for the preparation of CUR micelles able to treat ovarian (Abouzeid et al., 2014; Sarisozen et al., 2014) and colon (Abouzeid et al., 2013) cancer cells *in vitro* (Abouzeid et al., 2014) and *in vivo* (Abouzeid et al., 2013; Sarisozen et al., 2014), conferring synergistic effect to PTX- (Sarisozen et al., 2014) and DOX- (Abouzeid et al., 2013) based therapies.

Micelles composed of PEG-DOX conjugate were also prepared by self-assembly and proposed in a combination therapy for cervical cancer *in vitro* and hepatic cancer in xenograft model with favorable biodistribution and accumulation in toumors (Zhang et al., 2016).

The encapsulation of CUR into PX-based micelles was found to be highly toxic towards ovarian (Saxena & Hussain, 2013) and HeLa cancer cells (Sahu et al., 2011), while in association with RV, show a synergistic effect with DOX for the treatment of ovarian cancer and cardioprotective effects in mic (Carlson et al., 2014). The same effect was recorded by a RV-Q combination in ovarian tumors (Cote et al., 2015).

RV was proposed as therapeutic agent for breast cancer (Wang et al., 2015b), and glioblastoma (Kim et al., 2015a) treatment after encapsulation in PEG-PCL micelles bearing α-tocopheryl units (Wang et al., 2015b) or apolipoprotein obtained by recombinant DNA technique (Kim et al., 2015a).

Finally, some key literature data involve the use of micelles based on synthetic PEG-PLA (Sun et al., 2014a) or naturally occurring casein (Haratifar et al., 2014a,b) for the efficient EGCG delivery in pancreas (Sun et al., 2014a) and colon cancers *in vitro* (Haratifar et al., 2014a) and *in vivo* (Haratifar et al., 2014b).

## Carbon nanostructures and nanohybrids

Carbon nanostructures are a class on nanoscaled materials showing different sizes and shapes widely explored for use in biomedicine (Bhattacharya et al., 2016). Among them, carbon nanotubes (CNT) and graphene (G) gained a significant interest in the scientific community, due to their superior physico-chemical properties, large surface area available for interaction with biologically active agents, low cost, and ability in negotiating biological barriers (Bianco et al., 2005; Feng & Liu, 2011).

CNT are condensed benzene rings composed of sp^2^ carbons rolled up into a tubular structure with a single (SWNT) or multiple layer (MWNT) (Lin et al., 2004; Liu et al., 2009). They undergo membrane penetration and cell internalization through a spiraling or winding motion as well as through strong interactions with various proteins (Zhang et al., 2014b). Graphene (G) is single-layered six- sp^2^-carbon atom organized in a two-dimensional honeycombed network (Rao et al., 2009; Wang et al., 2011), up taken by cells via direct penetration events (e.g. endocytosis) or energy-dependent pathways (Peng et al., 2010). In biomedicine, G-based delivery vehicle is mainly obtained by using the oxidized derivative of G, named graphene oxide (GO), since the presence of abundant functional groups (epoxy, hydroxyl, carboxylic groups) has led to a surge of important potential in drug loading and delivery (Makharza et al., 2013).

The suitability of CNT and GO as drug carrier in cancer therapy is related to their pharmacokinetic profiles. CNT and GO distribute quickly throughout the body regardless of the administration route, accumulate in kidney, stomach and bone (CNT) or lungs, liver, and spleen (GO), and are excreted via urine and biliary pathways (Wang et al., 2004; Chaudhuri et al., 2010; Kiew et al., 2016). Nevertheless, the toxicity concerns dramatically reduce the potential application, mainly in the case of CNT (Lam et al., 2006), due to the fiber-like structure causing inflammation, fibrosis, granulomas, and necrosis, as a consequence of strong interference with the cellular redox state (Van Berlo et al., 2014). GO show improved biocompatibility features, even if cytotoxic effects can arise from impurities produced during synthesis or generation of intracellular ROS at the edge/internal defects (Bagri et al., 2010; Ambrosi et al., 2012).

The whole of the toxicity drawbacks can be successfully overcome by covalent or non-covalent surface functionalization with biocompatible hydrophilic compounds, mainly polymeric materials, with the generation of carbon nanohybrids (Spizzirri et al., 2015b).

In the literature, several different CNT and GO delivery vehicles for improved cancer therapy are reported (Spizzirri et al., 2015a), while only few examples of polyphenol carrier are available. To this regard, an interesting approach involves the reduction of GO by polyphenols and the simultaneous establishment of π interaction between the two species (Kim et al., 2015b). The resulting nanocarrier, obtained by using tea polyphenol extract (Abdolahad et al., 2013) or RV (Gurunathan et al., 2015), show high antiproliferative activity against colon and ovarian cancer cells, respectively.

In the case of CNT, although some researches report on polyphenol nanocarriers based on pristine CNT (Cirillo et al., 2011), biologically relevant materials are obtained only when they are coated with polymeric materials, such as GL and PMAA or PCL due to the toxicity problems above discussed (Table 1). By this approach, lung and hepatic cancer can be effectively treated by loading tea polyphenols onto PCL-MWNT nanoformulation prepared by electrospinning (Shao et al., 2011).

A different approach involves the preparation of functional nanohybrids, where the polyphenol is covalently attached to the polymeric counterpart by radical reaction. More in detail, CA was conjugated to GL and the obtained polymer therapeutic employed as coating for MWNT (Cirillo et al., 2013b; Castro Nava et al., 2016), while Q was employed in a one-step radical polymerization process in the presence of MWNT and methacrylic acid (MAA) (Cirillo et al., 2013a; Vittorio et al., 2014a). This approach was found top drastically enhance the anticancer activity of both flavonoids by means of improved stability and cell uptake features on HeLa cancer cells (Cirillo et al., 2013a,b). Interestingly, CA and Q nanohybrids can be employed in combination therapy with radiotherapy on prostate cancer (Castro Nava et al., 2016), and CP chemotherapy (Vittorio et al., 2014a) on neuroblastoma, showing considerable synergistic effects in both cases.

## Magnetic nanoparticles

Magnetic nanoparticles (MNPs) have shown promissory results as drug delivery nanosystems for anti-inflammatory, antitumor, or regenerative therapies. The main advantage of MNPs as carrier nanosystems is related to the possibility of being remotely directed toward a target tissue or organ by means of a magnetic field gradient (Riggio et al., 2014). Additionally, magnetic nanoparticles can be irradiated with an alternate magnetic field in the radiofrequency range to produce heat, increasing the possibility of synergic therapeutic action (Hernández et al., 2010; Asín et al., 2013). Both types of magnetic interactions (remote actuation and heating) require the MNPs to have an acceptable cytotoxic profile for *in vivo* applications. Moreover, a careful control of the magnetic properties such as magnetic moment and magnetic anisotropy must be provided for optimal performance. These properties are best realized when the magnetic colloids contain MNPs that are chemically stable and inert, and a narrow size distribution (Lima Jr et al., 2013).

There have been recently several attempts to use MNPs as carriers of polyphenols due to their known antitumor activity. *In vitro* tests using CUR conjugates as a cytotoxic agent on breast (Wani et al., 2011), glioma, and Caco-2 cells (Manju & Sreenivasan, 2011) have reported promising results, while nanoparticles prepared in the presence of PX show high anticancer activity with favorable pharmacokinetics and negligible immunogenicity and toxicity in pancreas xenograft models (Yallapu et al., 2013). MNPs were also prepared by coating with DEX–CA conjugate (Vittorio et al., 2014b) and reduction/chelation method with EGCG, and the resulting nanomaterials found to be effective on pancreas (Vittorio et al., 2014b) and colon cancer both *in vitro* (Vittorio et al., 2014b; Xiao et al., 2015) and *in vivo* (Xiao et al., 2015).

Furthermore, the *in vitro* cytotoxicity of Q delivered to SMMC-7721 tumor cells by Ni nanoparticles has been reported to operate on a synergistic effect between the Ni nanoparticles (increasing cell permeability) and the therapeutic Q (Guo et al., 2009). Although the magnetic properties of Ni particles were not exploited in this work, the potential uses of this property in addition to the synergistic effects described are obvious.

There are few reported works on polyphenols-delivery *in vivo* using nanoparticles as carriers, but it is currently accepted that the main limitations for any nanoparticle-based system such as non-specificity and short systemic circulation times also apply to polyphenol therapeutic strategies (Xiao et al., 2015).

## Conclusions and perspectives

The research for novel and effective therapeutic protocols for fighting cancer is still a challenge for researchers in different fields, from chemistry, biology, and physics to nanotechnology, engineering, and medicine. An overview of the literature clearly highlights the need to work in a multidisciplinary context to reach the expected results.

In oncology, naturally derived products such as polyphenols have a long-time history of promises and encouraging preliminary results, but their real applicability is still debated, since most of the *in vitro* and *in vivo* results have been not transferred to clinic. While it is clear that a therapeutic protocol for cancer treatment cannot be exclusively based on the employment of polyphenols, they can be proposed in combination with standard gene-, chemo-, and/or radio- therapies by virtue of the strong synergism found in different tumor models. Similarly, the wide range of nanomaterials available for the development of multiple delivery vehicles able to provide effective and non-toxic cancer treatment opens new opportunities for the clinical applicability of polyphenols.
